# Antarctic environmental change and biological responses

**DOI:** 10.1126/sciadv.aaz0888

**Published:** 2019-11-27

**Authors:** Peter Convey, Lloyd S. Peck

**Affiliations:** British Antarctic Survey, NE2C, High Cross, Madingley Road, Cambridge CB3 0ET, UK.

## Abstract

Antarctica and the surrounding Southern Ocean are facing complex environmental change. Their native biota has adapted to the region’s extreme conditions over many millions of years. This unique biota is now challenged by environmental change and the direct impacts of human activity. The terrestrial biota is characterized by considerable physiological and ecological flexibility and is expected to show increases in productivity, population sizes and ranges of individual species, and community complexity. However, the establishment of non-native organisms in both terrestrial and marine ecosystems may present an even greater threat than climate change itself. In the marine environment, much more limited response flexibility means that even small levels of warming are threatening. Changing sea ice has large impacts on ecosystem processes, while ocean acidification and coastal freshening are expected to have major impacts.

## Teaser

Climate change threatens Antarctica.

## INTRODUCTION – CLIMATE CHANGE IN ANTARCTICA

Antarctica excites the human imagination, be it the vast scale, environmental extremes, giant icebergs, awesome mountain ranges and vistas, or its charismatic wildlife. At the same time, it is central to Earth’s climate and oceanic circulation systems. While the explorers of the “heroic age” collected still vital samples and data, scientific study mostly only commenced after the Second World War and particularly with the International Geophysical Year (IGY) of 1957/8. Some parts remain biologically unsurveyed. Since the IGY, some parts of the continent, particularly the Antarctic Peninsula and Scotia Arc, have faced some of the most rapid environmental changes anywhere. This includes being one of the most rapidly warming regions globally, although much of this warming is underlain by regional rather than global processes (*1*, *2*).

Antarctica was key to the discovery of the stratospheric ozone hole, a consequence of anthropogenic atmospheric pollution, sparking global concern about the potentially harmful effects of ultraviolet (UV)–B radiation to biological, including human, systems (*3*). This led to the rapid negotiation and implementation of the Montreal Protocol, controlling the emission of the responsible chemicals. The ozone hole is suggested to have been one contributing factor currently protecting the main body of the Antarctic continent from the warming impacts of global climatic change (*3*–*6*, *7*). As long as the Montreal Protocol is adhered to, the ozone hole is predicted to repair over the next century, with the first clear evidence of this being reported recently (*8*–*10*).

Since around 2000, the strong atmospheric warming trend along the Antarctic Peninsula has paused, although it is predicted to resume (*11*). Over the next century, the entire continent is expected to start to see climatic changes comparable to those recorded to date along the Antarctic Peninsula (*12*, *13*). The predicted “filling” of the ozone hole is likely to provide further positive feedback to this process. Even with this level of change, the interior of the continent will remain far below zero and thus biological impacts are unlikely to be important. In coastal regions, summer air temperatures are already close to freezing, and warming will have far greater biological relevance, leading to increased melt and ice-free area especially around the Antarctic Peninsula (*14*). Globally, despite continuing increase in atmospheric CO_2_ concentration, there has been a recent slowdown in the rate of warming. This may be due to a redistribution of heat within the atmosphere-cryosphere system (*15*), with the reduction in atmospheric heating almost equating in energy terms to the contemporaneous increases in ice melting.

Seasonally ice-covered lakes can be particularly sensitive to environmental change and magnify the warming seen in air temperature (*16*, *17*). In the maritime Antarctic, warming and changes in precipitation have the most important influences, with increased biological production driven by reduced ice cover and mixing in the water column driven by surface exposure to wind. Some lakes contain indicators of changes in other environmental variables, such as increased salinity due to drier conditions and greater evaporation resulting from a change in prevailing wind direction (*18*). The negative impacts of consistently drier conditions are also apparent in changing patterns of moss abundance and health in parts of the continental Antarctic coastline (*19*).

The physical scale of Antarctica and wide variation in physical geography from the chronically cool and damp sub-Antarctic islands to the remote and high-altitude inland ice plateau and mountain ranges mean that there is no single description of its environmental conditions. The continent lies at the end of a range of global gradients in physical environmental variables, although its marine and terrestrial environments contrast in their thermal stability and rates of variation (*20*, *21*). The continent and its surrounding ocean and islands have been a focus for studies of the ecology, physiology, and, now, omics of life at extremes (*21*–*26*).

Warming in Antarctica and the Southern Ocean has not been uniform. Many continental regions have not exhibited significant change over the past century. In contrast, in some parts of the Antarctic Peninsula, annual mean air temperatures rose by 3°C or more between 1950 and 2000 (*1*). Sea temperatures in the Bellinshausen Sea to the west of the Peninsula increased by 1°C over the same period (*27*), accompanied by large-scale sea ice loss and the recession of coastal glaciers and ice shelves (*28*). Despite the recent pause in atmospheric warming over the Peninsula, coastal ice is still receding, and oceanic systems remain in flux (*29*). Recent marked decrease in overall Antarctic sea ice extent to record minimum levels [e.g., (*30*)] may be an important indicator of the onset of a negative trend [e.g., (*31*)] or of a tipping point being crossed (*32*, *33*).

Factors expected to affect Southern Ocean species arise predominantly from three major areas, increased temperature, altered sea ice [and iceberg scour in benthic habitats; (*34*)], and ocean acidification. The role of the cold Southern Ocean as a global atmospheric carbon sink has been highlighted, especially since the 2000s (*35*, *36*), exacerbating the challenge of acidification. Further factors, including salinity/freshening (*22*, *37*, *38*) and low oxygen levels (*39*), have potentially large impacts in coastal, and especially fiordic, environments (*21*, *40*–*43*). These factors often do not operate in isolation but rather synergistically, additively, or antagonistically.

Much attention is given to the ambitious target of the “Paris Agreement” to limit mean global warming to 1.5°C, even more challenging than the Intergovernmental Panel on Climate Change’s (IPCC’s) most conservative scenario limiting to a 2°C increase, by the end of this century. However, current trends of global temperature increase, sea level rise, and sea ice and land ice loss fall at the upper end of the IPCC’s more pessimistic scenarios. It is difficult to place the polar regions in the context of the Paris Agreement, given the widely recognized “polar amplification” of global change rates, especially when polar-specific local and regional effects are taken into account (*44*). However, the IPCC’s September 2019 Monaco statement (www.ipcc.ch/2019/09/25/srocc-press-release/) emphasized the important role of the oceans and cryosphere in global climate and responses to change.

The 20th century Antarctic Peninsula warming already exceeds the global two-degree target (*1*, *45*). Nevertheless, studies have sought to differentiate between the worst- and best-case scenarios (*46*), highlighting that, with appropriate coordinated global political will and action, human impacts on the global climate system may yet be controlled and mitigated to avoid the worst outcomes, albeit over multicentury time scales. With this context, this review provides a wide-ranging synthesis of the climatic and other environmental challenges facing Antarctica and the Southern Ocean, of the impacts these changes are already or will in the future impose on their biota and ecosystems, and of the biological responses already entrained or predicted.

## BIODIVERSITY PATTERNS

### Terrestrial

Less than 0.5%, and possibly as little as 0.18%, of Antarctica’s area is seasonally ice- or snow-free today (*47*, *48*), and most terrestrial ecosystems are effectively small “islands in the ice” (*49*), surrounded and isolated by solid rather than liquid water. Despite this, various terrestrial ecosystems are represented [see (*50*) for overview], whose biological complexity is largely driven by liquid water availability (*51*). The continental interior ecosystems include frigid deserts, nunataks, mountain ranges, and associated boulder/scree fields. With greater water availability in the coastal oases of the continental margin, and even more so along the western coast of the Antarctic Peninsula, these regions are characterized by cryptogam-dominated fellfields. The sub-Antarctic islands, with notably different levels of seasonality and chronically cool rather than extreme conditions, are generally well vegetated and more diverse.

The antiquity of most extant Antarctic terrestrial diversity (*52*) provides a long time scale for evolutionary divergence. For instance, microarthropod communities on some nunataks are separated by only tens of kilometers in Victoria Land but have been isolated and appear to have diverged on multimillion year time scales (*53*). The possibility of genetic homogenization and irreversible loss of genetic diversity should these ecosystems become linked through ice recession or other mechanisms has also been recognized (*54*) and provides a major conservation challenge (*55*, *56*). Very high levels of species endemism, often at or below the much smaller geographic scale of the Antarctic Conservation Biogeographic Regions (ACBRs) (*47*), are typical of many Antarctic terrestrial biota (*57*–*59*). This again creates unique spatial conservation and management challenges for the continent and surrounding ocean (*60*–*63*).

The Convention on Biological Diversity defines “biological diversity” as “the variability among living organisms from all sources including, *inter alia*, terrestrial, marine and other aquatic ecosystems and the ecological complexes of which they are part; this includes diversity within species, between species, and of ecosystems” (*64*). Antarctic terrestrial species richness is generally low (*50*, *65*), although this increasingly does not appear to be the case for microbial and viral diversity (*66*–*68*). The general simplicity of Antarctic terrestrial ecosystems makes them attractive model systems for studies of responses and sensitivities to environmental variability and change.

### Marine

Marine diversity, particularly of benthic groups, stands in complete contrast to that on land and has been described as second only to coral reefs globally (*21*, *69*, *70*). More than 8000 invertebrate species have been described from the Southern Ocean (*70*), with the total number estimated to be 17 to 20,000 (*71*). Several factors might drive estimates even higher, including low levels of sampling in parts of the Southern Ocean and poor sampling quality and effort in some others (*69*, *72*). Molecular techniques are identifying previously unknown cryptic species, and species not previously sampled, from metagenomic or environmental DNA surveys.

When evaluating Antarctic marine biodiversity, several factors should be, but rarely are, taken into account (*21*). Two such factors are that there is no year-round ice-free intertidal on the continent and that the continental shelf is deeper than for any other continent. Ice growing on the intertidal and at shallow subtidal depths severely limits diversity inhabiting these areas (*73*, *74*). In temperate and tropical areas, in contrast, intertidal and shallow sites contain high diversity. The Antarctic continental shelf depression is due to the 26.5 gigatons (Gt) of ice present in the Antarctic ice sheets (*75*), with the outer edge of the shelf at 800- to 1000-m depth compared with around 200 m for other continents. Thus, although Antarctica’s shelf accounts for over 10% of global shelf area, it has only 2.1% of the global ocean area shallower than 200 m (*21*). Biodiversity (as numbers of species) generally declines with depth, usually explained as an area of available habitat effect (*76*). The relatively high levels of Antarctic marine biodiversity are therefore even more unexpected.

Antarctic marine biodiversity is not consistently high across taxonomic groups. Thus, there are no representatives of the globally common brachyuran crabs, but spider crabs, the Majoidea, and lithodid king crabs are present (*70*). Among the cartilaginous fish, there are no sharks or rays, but there are skates. The reason that sharks do not inhabit the coldest waters is not clear, but it is hypothesized that the need to swim to ventilate gills in most species gives an unsustainably high energy requirement when combined with the reduction in power output from muscles as temperature falls (*21*). A second hypothesis is that high levels of the metabolic waste products urea and trimethylamine N-oxide may affect osmotic gradients in cells negatively at very low temperatures (*77*). Other groups of fish are also absent, including salmonids, and diversity is dominated by one group, the notothenioids, which account for over 70% of fish species in the Southern Ocean but are not common elsewhere (*78*). Some groups, such as gastropod snails and bivalve molluscs, are poorly represented with four times fewer species per unit area than at lower latitudes. Other groups, however, such as isopod and amphipod crustaceans and the globally very diverse polychaete worms, have more species per unit area on average than at lower latitudes, and pycnogonid sea spiders have over twice as many species as the global average (*21*, *69*). The high diversity in sea spiders is thought to be partly because they replace the brachyuran crabs as predators. They are also an example of gigantism at low temperature, along with other groups including isopods and amphipods, and this is related to both low temperature reducing metabolic rates, which lowers the cost of maintaining tissues, and higher levels of dissolved oxygen at low temperatures (*79*, *80*).

**Fig. 1:**
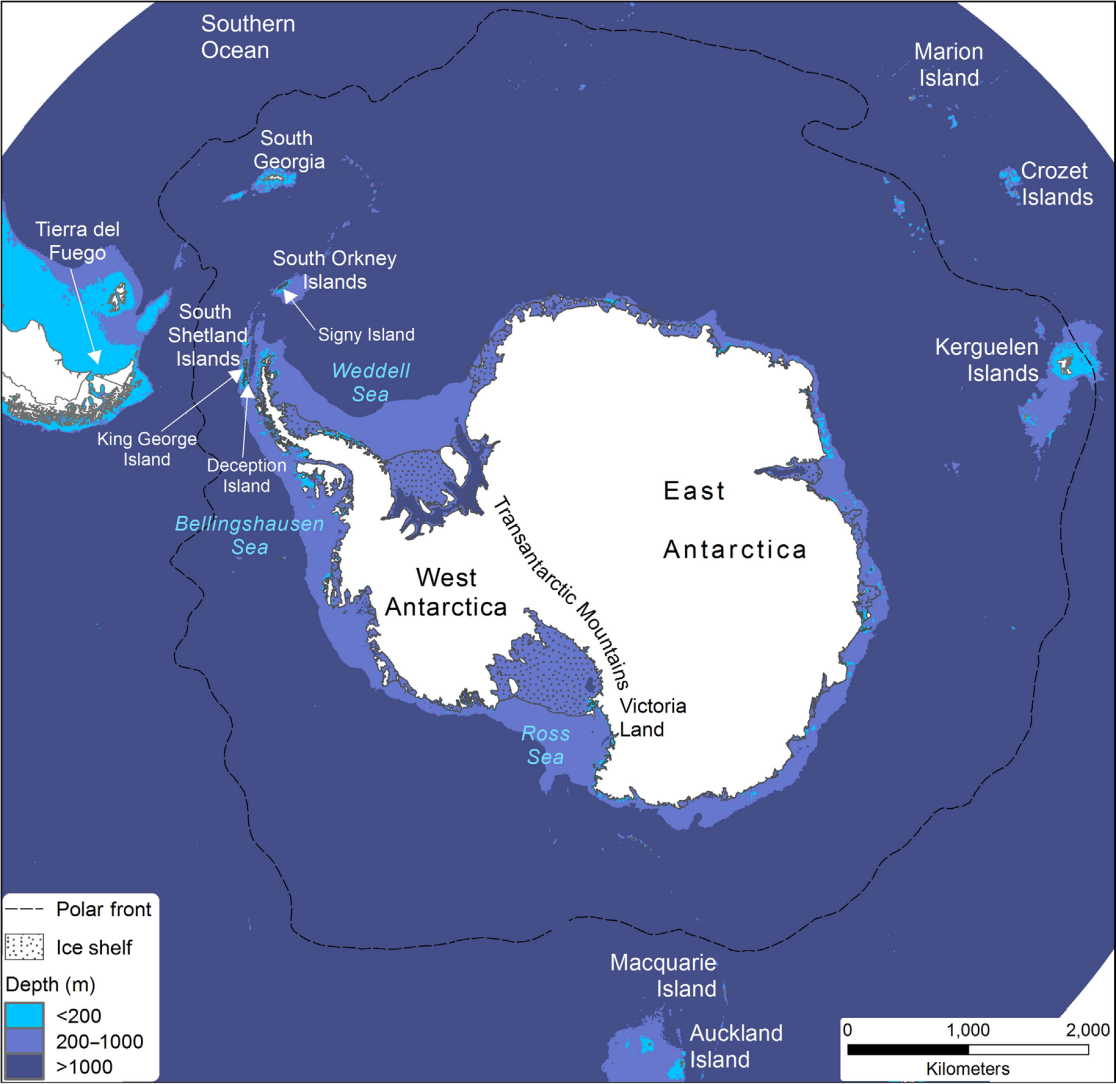
Map of Antarctica, showing locations mentioned in the text, and the Southern Ocean, showing ice-covered and ice-free areas shallower than 200 m, 200- to 1000-m depth, and deeper than 1000 m [modified from (*21*); image provided by P. Fretwell, British Antarctic Survey].

Many factors underlie the high biodiversity in the Southern Ocean, including high environmental heterogeneity, isolation, and age (*21*). There is environmental heterogeneity from small to large spatial scales that is caused by the following: variation in nutrient dynamics; variation in summer light availability, influenced by latitude and factors such as ice cover and sediment load; and variation in salinity and glacier runoff that also affects sedimentation and turbidity. Habitats in sea ice vary over small spatial scales due to vertical light gradients and strong salinity changes over both spatial and temporal scales [e.g., (*81*)]. Antarctica’s isolation has allowed many new species to evolve in the absence of competition from lower latitudes. In addition, having the largest geographic separation from any other continent, it is the only continent that lacks continental shelf connectivity with another continent (Fig. 1) (*21*). A further factor is that, during glacial cycles, areas where life could persist contracted into isolated refugia and then expanded again during warmer periods. The isolation allowed new species to arise and then mix as they came into contact again when conditions warmed, and this mechanism driving speciation in Antarctica has been called the biodiversity pump (*82*).

## SENSITIVITY TO ENVIRONMENTAL CHANGE

### Terrestrial

Polar terrestrial ecosystems are recognized as sensitive to environmental change (*83*–*85*). While global perceptions of the consequences of many aspects of environmental change are negative, the responses in Antarctic terrestrial ecosystems to warming in particular may be positive. This possibility arises from the combination of warming trends in parts of Antarctica, leading to more cumulative energy being available to biota, both in terms of the absolute positive temperatures achieved and in cumulative degree days (*45*). There is also more snow and ice melt that releases liquid water and expands the area available for colonization (*14*, *51*, *55*, *86*). Relaxation of the current environmental limits imposed by low temperature and desiccation could encourage increased productivity, population growth, and expanded local distributions. However, several other outcomes are also possible. For instance, where increased melt leads to exhaustion of the source supply, affected areas will become less, not more, suitable for biological communities [e.g., (*19*)]. Changes in nutrient supply, e.g., nitrogen derived from marine vertebrates (*87*) or step changes in key ecosystem services such as decomposition driven by new (usually anthropogenically introduced) community members (*88*), will likely favor stronger competitors for nitrogen in the native community (such as grasses over mosses) (*89*). Other circumstances in which altered stress levels have been observed or predicted include changes in radiation levels (increased/decreased cloud cover or ozone hole–associated UV-B receipt) (*90*), local cooling (*91*), and changes in frequencies of freeze-thaw events (*56*, *92*), wind patterns (*18*), or precipitation (*14*, *93*).

Antarctic terrestrial ecosystems are not entirely isolated from those of the rest of the world. A commonly predicted consequence of environmental change is that native species distributions will change, and that non-native species will invade. Both of these events could occur even in the absence of environmental change, the latter, in particular, through human assistance (see the section on “Non-native species”). Where environmental change is an ameliorating influence, it is likely to act in synergy with human activity, increasing the probability of successful transfer and colonization of non-native species (*94*).

Abiotic factors—physicochemical environmental conditions—are currently considered the predominant drivers of ecosystem processes in more extreme environments such as those of the Antarctic continent. This is consistent with the generally adversity-selected life history strategies of the terrestrial biota of the Antarctic Peninsula and continent (*95*, *96*). Nevertheless, this has rarely been tested explicitly, and autecological studies of Antarctic terrestrial species are very rare. Some recent studies in both the Antarctic Peninsula and Victoria Land suggest that biotic interactions may play a greater role than previously suspected even in some more extreme environments (*97*, *98*). With environmental amelioration, the importance of biotic factors including competition, herbivory, and predation will likely increase, as is the case on some of the sub-Antarctic islands (*99*). There is also concern about the potential for increased movement/incidence of disease in vertebrates (*100*), or that lower latitude features such as “red tides” (harmful cyanobacterial blooms) may spread to affect parts of the Antarctic as conditions become more favorable (*101*).

Despite the well-documented climate change trends particularly along the Antarctic Peninsula, unexpectedly, few explicit studies of biological responses are available from natural ecosystems. The best-documented have been local population increases in the two flowering plant species native to the maritime Antarctic (*102*–*104*) and the inference of increased frequency of successful seed set (i.e., sexual reproduction) in concert with this (*105*). Warmer temperatures and increasing liquid water availability improve growth of established plants, seed maturation, germination, and establishment. Continental Antarctic soil nematodes show responses to both climate trends and to rare melt events (*86*). Despite anecdotal observations of rapid development of the dominant cryptogamic vegetation of the maritime Antarctic, including rapid colonization of newly exposed ice-free areas, the only study that appears to document this robustly is that of (*106*).

Biological processes tend to operate at the individual and microhabitat scale. In the Antarctic terrestrial environment, this means that variability at the scale of millimeters to meters (*92*). It is therefore challenging to confirm whether the biota is sensitive to macroclimatic variables at the resolution used in most climate change studies. Long-term patterns (seasonal/annual) of variation in water relations of terrestrial arthropods in the maritime Antarctic are consistent with seasonal climate variation and overall climate trends, showing that they are sensitive and responsive to changes of the magnitude that are already being seen (*107*).

### Marine

Antarctic marine environments are among both the most variable and the least variable globally. They are thermally very stable, with the highest latitude sites varying between −1.9°C (the freezing point of seawater) and −0.5°C annually (*108*) and the most variable sites varying by over 4°C (*109*). In contrast, seasonal light variation drives large changes in sea ice cover. These factors produce among the shortest summer phytoplankton blooms globally, and nearshore blooms are among the most intense reported anywhere, with chlorophyll concentrations at times exceeding 50 mg Chl m^−3^ (Rothera Time Series; www.bas.ac.uk/project/rats/).

Organisms can respond to altered environments at process scales from the molecular to the ecosystem, and responses vary with the temporal and spatial scales of change (*21*, *110*). At the cellular level, biochemical buffering dominates responses. Above this comes gene expression and then plasticity of the phenotype via physiological flexibility. These processes buffer changes over hours to weeks. Above this, alterations of gene frequencies, selection of individuals in populations, and behavioral modifications are important. Over years to centuries, phenotypic plasticity, evolutionary genetic responses, and speciation are key (*110*–*113*). These mechanisms determine survival of environmental changes and cascade to responses at the largest scales in terms of ecological interactions, migration, distribution changes, and, eventually, ecosystem alteration and stability (*21*, *114*). There is consensus that the most important responses for survival of climate change and maximizing individuals to species fitness are phenotypic plasticity, especially through acclimatization of physiological processes, and modification of the population gene pool, genetic adaptation (*21*, *110*–*112*). Species with short generation times (days to months) respond primarily through genetic modification and require little phenotypic plasticity. Species with long generation times (years to decades), e.g., most Antarctic marine invertebrates and fish, depend on phenotypic plasticity to survive long enough for adaptation processes to take effect (*21*, *65*, *110*–*112*).

**Fig. 2:**
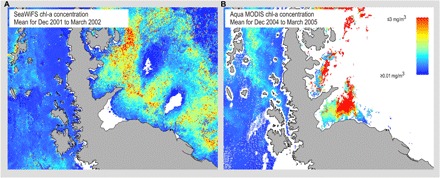
Satellite images of the area surrounding the original Larsen B ice shelf. (**A**) Ice-covered area in 2000 before its collapse, and (**B**) in March 2004/5, showing chlorophyll (chl) concentrations from the dense phytoplankton bloom that was present in the newly exposed area (white areas were sea ice covered and gave no signal) [from (*266*)]. SeaWIFS (Sea-Viewing Wide Field-of-View Sensor) is a satellite borne sensor for measuring Chlorophyll in surface ocean waters; MODIS (Moderate Resolution Imaging Spectroradiometer)is an instrument monitoring the Earth’s atmosphere, ocean, and land surface with a set of visible, NIR, MIR, and thermal channels run by NASA.

Reductions in coastal ice, sea ice, and iceberg scour increases have primarily occurred along the Antarctic Peninsula. Responses in the biota have mainly been assessed via ecological impacts [e.g., (*115*–*119*)], with both positive and negative impacts. Loss of coastal glaciers and ice shelves has opened up new areas for biological productivity (Fig. 2) (*117*). The creation of new large areas of seabed combined with new phytoplankton productivity in Antarctica may be the second largest natural feedback globally sequestering carbon and slowing warming (*117*). This sequestered carbon has been called “Blue Carbon” (*34*). Alongside this, research has started to address recolonization and succession processes in benthic environments [e.g., (*120*)]. Reported negative responses include reduced krill numbers and altered distribution with decreased sea ice [e.g., (*121*)], with knock-on impacts on other major elements of the Southern Ocean food web including penguins, albatrosses, seals, and whales (*41*, *122*–*124*). Increased iceberg activity locally destroys benthic communities (*116*), limiting growth and carbon sequestration (*34*, *125*). The slow growth of many benthic species means that recovery from substantial iceberg scour is a much slower process than seen in communities at lower latitudes from analogous disturbances such as trawling (*21*, *116*).

Many laboratory studies have focused on the effects of elevated temperature on Antarctic marine species. These include studies on fish [e.g., (*126*, *127*)], molluscs [e.g., (*128*–*131*)], echinoderms (*132*, *133*), amphipods (*22*, *134*), isopods [e.g., (*135*, *136*)], and sponges (*137*). There have also been assessments of elevated temperature impacts using larger-scale approaches, aimed at quantifying multispecies, community, ecosystem, or overall biodiversity level responses [e.g., (*24*, *138*, *139*)]. One study has led the field globally in conducting experimental temperature manipulations in situ on the seabed (see section on “Manipulation studies”) (*140*, *141*). The key result is that Antarctic marine species are poor or very poor at surviving environmental warming. This vulnerability was first identified in the 1960s [e.g., (*142*)] and has been summarized in recent reviews (*21*, *113*). Antarctic marine species appear to have similar physiological limitations to cope with warming as tropical species, and both are much more sensitive than temperate species (*21*, *143*).

Another area of concern in Antarctic marine systems is ocean acidification. Data here are conflicting, with some studies showing large acidification impacts on, e.g., pteropods (*144*, *145*), and early developmental stages in other species [e.g., (*146*, *147*)]. However, several other studies have shown Antarctic species cope well in low pH [e.g., (*148*–*150*)], especially when long exposure periods are used that allow animals to acclimate their physiology [e.g., (*133*)].

Other marine environmental factors predicted to change include salinity, oxygen, and sedimentation. Warming melts more land-based ice, which increases freshwater runoff. This causes a general freshening of seawater, especially in coastal sites, with most impact in partially enclosed fjordic systems (*41*). Extensive ice loss has occurred over recent decades along the Antarctic Peninsula, and currently, the West Antarctic ice sheet is losing mass rapidly. It lost around 250 Gt/year between 2009 and 2017, three to four times more than the rest of Antarctica combined (*151*). The large volume of fresh water discharges mainly into a relatively limited area, the Amundsen Sea. There are currently no reports of the impact of this freshening on the marine biota of the region. A general increase in ice loss, and hence freshwater and sediment release, is predicted around Antarctica, which could have massive impacts on local seabed communities (*43*).

Biodiversity is heavily affected by sediment load in some Arctic fjords, where strong gradients in numbers of species and biomass occur in relation to turbidity and inorganic deposition (*152*, *153*). Similar, but less intense, effects have been documented on King George Island (*154*), while high freshwater and sediment inputs have been associated with mass mortality events in krill (*40*). Antarctic fjords are hot spots for biodiversity because they are much more diverse than the seabed elsewhere (*155*), although many parts of the Antarctic coastline do not have fjords that are open for biodiversity colonization.

Oceanic warming will reduce oxygen levels available for organisms as oxygen solubility and concentration increase as temperature decreases (*156*, *157*). The high concentration of oxygen in Antarctic waters is likely a problem for many species due to damage in cells from reactive oxygen species (ROS), and Antarctic marine species, in general, have very strong molecular defenses against ROS damage (*158*). Warming in the Southern Ocean is, therefore, likely to have conflicting impacts. Negative impacts include increased metabolic rates and reduced oxygen availability, thus affecting abilities to produce energy for work without using anaerobic processes that produce toxic end products (*113*, *159*, *160*). This should set temperature limits for species in warming environments (*161*, *162*). However, although this mechanism does set limits in some laboratory regimes [e.g., (*163*)], support for the universality of oxygen limitation is limited, and it seems that different mechanisms set temperature limits for different species and also under different rates of warming (*21*, *24*). Positive impacts include the following: a small warming should decrease ROS damage and reduce the need for defenses. It should also reduce the challenge of making proteins at low temperature, which limits growth and development rates (*21*, *164*). There are, thus, factors driving in different directions in terms of the impact on Antarctic marine species of lower oxygen in a warming environment. Nevertheless, reduced environmental oxygen will be a challenge globally for marine species [e.g., (*165*)].

There is a prescient need for commitment to long-term multidisciplinary evaluations of environmental change and the responses of biota in terms of their distributions, physiologies, population genetic modification, and community and ecosystem structure and function. This is also needed to identify species and ecosystems that are vulnerable to change, both to predict future outcomes and to ensure that the best conservation practices are used. Both terrestrial and marine biodiversity are threatened by ongoing and predicted change. In both environments, warming is causing powerful impacts for organism survival, but beyond that, the major factors are mainly consequences of warming, and they are very different between land and sea. Cross-disciplinary research in Antarctica is urgently needed to assess how species are and can respond to environmental insults, knowledge that is crucial to predicting future impacts from (micro)environmental changes, distribution changes, population and species survival, and rapid alterations in ecosystem balance (sometimes termed tipping points) with the resultant consequences for services to society (*43*, *46*, *166*).

Biodiversity in Antarctica in both terrestrial and marine environments is viewed as being under threat, and among the most threatened anywhere, from future changes. Some factors are common to both, such as the impacts of warming and from alien invasions. Other factors differ, however, with water availability being a prime game changer on land and sea ice loss, iceberg scour, sediment load, and freshening being large factors for marine ecosystems. There is important recent recognition that different environmental factors altered by climate change can act in concert or synergistically, and studies are now being conducted on multiple factor effects [e.g., (*42*, *167*)]. In marine systems, these have demonstrated, e.g., that temperature has a stronger impact on organisms than acidification [e.g., (*149*, *168*–*170*)].

## MANIPULATION STUDIES

### Terrestrial

Experimental field manipulation studies, while very challenging in the harsh and remote Antarctic terrestrial environments, have been a primary means of modeling some of the predicted changes under environmental conditions and their impacts on species and communities [e.g., (*86*, *171*–*177*)]. Serious methodological limitations initially meant that the manipulated conditions failed to give good representation of predictions (*178*). A review of field studies that attempted to experimentally model ozone hole impacts (*179*) concluded that there was no overall consistency in the effects obtained through using a standard lamp augmentation methodology for imitating the changes in ozone hole–related UV-B receipt by terrestrial ecosystems.

Early studies confirmed that the dominant elements of typical terrestrial communities (microbial flora, bryophytes, and invertebrates) responded rapidly in terms of biomass, population density, and ground cover to the changes imposed (*180*–*183*). The development of methodologies with improved replication, more realistic manipulation of more environmental variables, and accurate microenvironmental monitoring has given these studies greater reliability (*172*, *177*, *184*–*186*). Nevertheless, recent reviews of manipulations applied in both polar regions (*175*, *176*) concluded that they rarely provide a good representation of predicted changes over the annual cycle and may even lead to changes opposite to those expected.

### Marine

There have been few experimental environmental manipulations in Antarctic marine systems, mainly through practical constraints and, in particular, ice scour. However, studies have assessed colonization of new surfaces and the development of seabed communities on settlement panels deployed for periods up to 22 years [e.g., (*187*, *188*)], and these have demonstrated generally very slow recruitment and growth interspersed with infrequent periods of more rapid colonization and growth. Even the most rapid rates are still much slower than the fastest reported in temperate and tropical sites, and growth and colonization are greatly reduced compared with warmer environments (*21*). Other studies deploying equipment at depths vulnerable to ice scour include environmental logging systems [e.g., temperature; (*189*, *190*)] and monitors of iceberg scouring activity itself (*34*). Possibly, the most ambitious study has deployed heated settlement panels to evaluate the effects of warming the seabed by 1° or 2°C for periods up to 2 years. Unexpectedly, a 1°C warming doubled growth rates, but a 2°C rise took some species to or beyond their limits (*114*, *140*). In situ manipulations have very large advantages over laboratory experiments, as many environmental factors remain natural. They can also be deployed for much longer, providing better simulations of future conditions than achievable in the laboratory.

## DIRECT HUMAN IMPACTS

Relatively few people visit Antarctica on a yearly basis—around 5000 national operator staff and approaching 50,000 tourists (*191*, *192*). Research activities are concentrated around the research stations in the South Shetland Islands and northern Antarctic Peninsula, as well as those in Victoria Land. There are no trading ports, native human populations, industrial developments, or trade routes. The Southern Ocean supports important fisheries that are regulated under the Convention for the Conservation of Antarctic Marine Living Resources (CCAMLR), a Convention of the Antarctic Treaty. Tourist operations primarily use smaller cruise ships and focus on a relatively small number of well-known locations, mostly in the South Shetlands and northern Antarctic Peninsula (*192*, *193*). Research staff and their support personnel typically spend longer periods based at a single location, while tourists participate in short landings across several locations in quick succession, spending far less time on land overall. National operators also land cargo at stations and field sites and support remote operations across the entire continent (*61*, *191*). The “stepping stone” nature of many logistic and tour vessel routes, visiting successive locations within or between Antarctic regions, exacerbates the risk of intra- and interregional transfer of both native and non-native organisms (*192*, *193*) (see below).

Direct human impacts provide a distinct set of threats over and above those associated with climate change. On land, human activity focuses on the very small land area that is ice free, predominantly near the coast where most research stations and visitor sites are located. These are the same areas in which terrestrial ecosystems are best developed and that host marine vertebrate breeding and molting concentrations. Thus, there is competition for access to and use of the very limited resource of ice-free land, with the result that it has recently been documented that an unusually large proportion is already affected by human activity (*194*, *195*).

The continuing impacts of historical marine exploitation and other industrial activities are felt in parts of the Antarctic, particularly the sub-Antarctic islands and the northern maritime Antarctic (*196*, *197*). On land, these primarily include the remains of onshore whaling stations, some of which were major industrial sites (*198*). Some, such as Grytviken (South Georgia) and Whaler’s Bay (Deception Island), have been stabilized and at least partially cleaned up and are now historical monuments, while others include increasingly dispersed debris and various types of pollution. These historical industries almost wiped out fur seals in the late 18th and 19th centuries, followed by the great whales in the 20th century. These major marine ecosystem disruptions leave us unable to reconstruct its original state, and the Southern Ocean ecosystem is still recovering from them. Before CCAMLR, there was almost uncontrolled overexploitation of a range of finfish species in various Southern Ocean regions, again with limited evidence of subsequent recovery [e.g., (*199*)]. Today’s active industrial fishing industry does not use land-based support facilities, although (along with national operator and tourism shipping operations) there is still the potential for both terrestrial and marine impacts resulting from accidents, shipwrecks, and associated pollution (*191*, *200*).

Studies quantifying direct human disturbance are rare [but see (*194*, *201*–*203*)]. Ships, research stations, and travel activities create chemical pollution, local dispersal of dust (affecting snow surface albedo), and on land can damage soil surfaces, vegetation, and freshwater ecosystems (*204*, *205*). Even human footfall can compress the soil structure and visibly damage vegetation and alter invertebrate community structure (*201*, *202*, *206*, *207*). Recovery from these disturbances may take many decades, with vehicle tracks and even footprints remaining visible [e.g., (*208*, *209*)].

Avoiding and mitigating damage require active education and adherence to existing procedures and advice, although there is a lack of investment in monitoring of either impacts or recovery (*210*). “Human footprint” assessments are now being carried out in ice-free areas [e.g., (*194*, *203*, *211*)]. These efforts are beginning to demonstrate previously unappreciated large-scale environmental modification (*195*), although this area of research is still in its early stages.

A specific example of the ongoing and unexpected consequences of previous human exploitation of Antarctic marine ecosystems has particularly important implications for some terrestrial ecosystems along the Antarctic Peninsula, and Scotia Arc is given by the recovery of Antarctic fur seals to at least preexploitation population levels. Although this recovery is centered on their main original breeding grounds on South Georgia, nonbreeding seals now occupy regions (South Orkney Islands and most of the western Antarctic Peninsula) where there is no evidence that they have occurred previously (*212*, *213*). This expanding range is leading to large-scale destruction of the typical, but fragile, terrestrial flora and faunas over accessible areas (*214*, *215*) and to extensive eutrophication of lake ecosystems (*216*, *217*). The scale of these impacts far exceeds any predicted consequences of climate change alone.

Marine pollution studies have assessed sewage outfalls from stations [e.g., (*218*)], heavy metal concentrations [e.g., (*204*, *205*, *219*)], and used animals as monitors for pollutants [e.g., (*220*, *221*)]. Humans have also acted as vectors for disease transmission in marine wildlife [e.g., (*222*)].

Direct impacts also come from structures and facilities. There are only a few sites compared with temperate and tropical regions that have experienced large-scale environmental modification from built structures and facilities. However, there is also relatively little accessible coastline, and it is estimated that over 50% of this has been affected in some way (*43*). Today, all built structures require environmental impact assessments and the minimization of environmental impact (Antarctic Treaty Environment Protocol; www.ats.aq/e/eia.html). However, there have been unintentional impacts in the past, and there is substantial effort being put into remediation, although not all restoration has the same efficiency or outcome (*191*, *223*, *224*). Station construction exerts considerable local disturbance, and station footprints affect a considerable proportion of available terrestrial area in parts of the continent (*203*). This is a continuing pressure, with major station (re)building and infrastructure construction projects at Italian, New Zealand, and U.S. stations in the Ross Sea; Chinese stations on the inland continental ice sheet; and Brazilian, Turkish, U.K., and U.S. stations in the Antarctic Peninsula. The future may see major changes to this situation, with ideas ranging from the building of CO_2_ sequestering process plants, taking advantage of Antarctica’s very low temperatures that are already close to the condensation point for CO_2_ in the middle of the continent in mid-winter, to the geoengineering of Antarctic glaciers to slow sea level rise (*225*).

## NON-NATIVE SPECIES

The physical isolation of Antarctic terrestrial and marine ecosystems, along with their harsh environmental conditions, has placed strong but not complete limitation on biological colonization by non-native biota (*226*–*228*). Human assistance provides a means to overcome these barriers (*94*). Although few studies have addressed the relative importance of natural and human-assisted colonization routes, data from some Southern Ocean islands (*229*, *230*) suggest that the latter has been responsible for at least 100× more species establishment events than natural processes in the centuries since their discovery. There are presently no known examples of natural establishment of new species on the Antarctic continent or Peninsula since human contact with the region, while the number of human-assisted events is increasing (*231*).

Very few non-native species have established on the Antarctic Peninsula and continent to date (*231*). Although the impacts of non-native species have thus far been small to undetectable, the potential danger is well demonstrated by introductions on many of the sub-Antarctic islands (*196*, *200*). When non-native species do establish, a form of “ecosystem engineering” may take place, for instance, through previously absent ecological/trophic guilds (e.g., new predatory or pollinating guilds) being introduced and step changes to ecosystem services taking place (*88*, *232*–*238*), as seen elsewhere on the planet. Some of these changes are likely to be irreversible, including the threat of local or even complete extinction of native endemic invertebrate species. Energy flow in native Antarctic terrestrial ecosystems is dominated by the microbial decomposition cycle (*239*). The introduction of grazing and predatory invertebrate guilds in synergy with climate change could generate a tipping point in function in these ecosystems, with currently unknown consequences.

Future distribution modeling approaches have started to be applied to both native and invading terrestrial biota in Antarctica. An example is the chironomid midge, *Eretmoptera murphyi*, a palaeo-endemic species from South Georgia (*240*), which was accidentally introduced to maritime Antarctic Signy Island in the 1960s. Detailed modeling, based on knowledge of the species’ physiological tolerances, confirms its ability to expand distribution considerably on Signy (*241*), while simple climate matching suggests that it would be capable of surviving in habitats that already exist in almost the entire length of the western Antarctic Peninsula (*237*). Similarly, the invasive grass *Poa annua*, already established on King George Island, has the potential to spread further south along the Antarctic Peninsula (*242*). Similar studies applied to native invertebrates come to the same conclusion. The native midge *Parochlus steinenii*, currently restricted to the South Shetland Islands, under both the IPCC Representative Concentration Pathway (RCP) 4.5 and RCP8.5 scenarios could occupy habitats along both the east and west coasts of the Antarctic Peninsula and, under the latter scenario, parts of the East Antarctic coastline should suitable transport opportunity occurs. The latter outcome also emphasizes concerns that the combination of climate similarity and human operational connectivity further compounds the risk of human-assisted transfer (*192*). The risk of microbial introductions has also been highlighted, but few data exist (*243*). The single recorded continental Antarctic establishment has been eradicated, and no confirmed establishments are known from Antarctic marine environments (*192*).

Historical vertebrate introductions to the sub- and peri-Antarctic islands involved grazing (rabbits, reindeer, sheep, mouflon, cattle, and pigs) and predatory (cats and rodents) mammals as well as aggressively invading plants. These led to widespread and marked impacts on native vegetation, ground-nesting birds, and invertebrates (*244*–*247*). Deliberate introductions are now prohibited, although accidental introductions of rodents cannot be discounted. Of the currently known non-native species in Antarctica, virtually all can most plausibly be linked with either national operations or historical exploitation industries (*191*, *196*, *200*).

Expensive and logistically committing exterminations of some introduced vertebrates have now been completed [e.g. (*247*, *248*)], and others are planned. For these efforts to be worthwhile investments of resources, continued attention and commitment to stringent biosecurity procedures are required. Other than the continental Antarctic grass eradication mentioned above, only two other eradications have been documented in the maritime Antarctic, both of flowering plants. These were of a single patch of the cosmopolitan grass *Poa pratensis* at Cierva Point (*249*) and a single plant of the Tierra del Fuego native *Nassauvia magellanica* on Deception Island (*250*), with the latter also highlighting the management challenge of separating a putative human assisted from a natural colonization event [see (*251*) for discussion]. The final removal of sledge dogs associated with field operations of several national operators took place in the mid-1990s. Remedial action applying to most plant and invertebrate species currently established in Antarctica, not to mention any future marine invading species, is likely to be impracticable. Mitigation responses must therefore focus on intensifying biosecurity actions to minimize the risk of further spread from already established locations.

Predictions are that environmental alteration under climate change will be conducive to the establishment of non-native species in Antarctic marine environments [e.g., (*252*)] as in the terrestrial environment. Marine biodiversity may be particularly at risk from non-native species invasions due to its long period of isolation and lack of competition from lower latitude species. Furthermore, ship activity—a major transport vector—has increased up to 10-fold since the 1960s [e.g., (*253*, *254*)]. There are currently no confirmed records of non-native marine species establishment around Antarctica. Only five free-living marine species have been reported from Antarctica (not established) that were potentially transported by anthropogenic routes (*192*): *Ulva intestinalis* (grass kelp or gut weed), *Hyas araneus* (great spider crab), *Bugula neritina* (brown bryozoan), *Ciona intestinalis* (vase tunicate), and *Ectopleura crocea* (pinkmouth hydroid). Given predicted future environmental changes and the ever-increasing human traffic, it is not a matter of if non-native species will arrive but when, and entraining processes to minimize these chances must be a very high priority.

The combination of increased human activity, including inter-regional logistical routes within Antarctica, and climate change will reduce barriers to invasion in both terrestrial and marine ecosystems (*94*, *192*, *255*, *256*). Accidental transfers of non-native species into Antarctica probably occur more frequently than is recorded. Some have established synanthropically (directly associated with human activity), usually within station facilities where they are protected from natural environmental stresses (*257*, *258*). While apparently not able to survive externally at present, this creates a pool of potential colonizers already present within Antarctica in terrestrial systems. In marine environments, a direct comparison does not exist, although non-native species established within “sea chests” on ships present an analogous risk.

## ANTARCTIC REGIONALIZATION AND FUTURE CONSERVATION CHALLENGES

The complex patterns of biogeographic regionalization now recognized have resulted in the definition of 16 distinct terrestrial ACBRs (*47*, *259*). These now form a fundamental basis to future conservation planning within the Antarctic Treaty System and have also resulted in new risks being recognized through human assistance with intracontinental movement of biota indigenous to different areas of the continent. Older observational and recent modeling studies have emphasized that these risks apply both within and between ACBRs (*192*, *242*, *260*). Many of these species likely have preadapted ecophysiological and life history characteristics that would support establishment in multiple ACBRs, as exemplified by the invading dipterans *E. murphyi* (*237*, *241*) and *Trichocera maculipennis* (*238*, *261*).

Clear and urgent commitment is required by national operators and funding agencies responsible for research across Antarctica to the establishment of continent-wide baseline survey and monitoring programs, backed by appropriate expertise (*210*, *262*), and of research into the status and impacts of known non-native biota. Commitment is also required, where practicable, to the rapid eradication of non-native species from known locations and, if not, the implementation of robust awareness and biosecurity measures to minimize the risk of further spread [see (*250*, *251*, *263*)].

## CONCLUSIONS

One of the founding Antarctic Treaty principles, reaffirmed in the “Santiago Declaration” of 2016 (*264*, *265*), is to ensure the preservation and protection of the Antarctic environment. Antarctica faces twin challenges from the multiple consequences derived from global environmental change and more local-scale direct impacts of human activity, and both need attention if this founding principle is to be achieved. Climate change is but one of the threats facing Antarctica in the next century and beyond, and some of the direct consequences of human activity, particularly those of historical marine exploitation, land use change, and biological invasions, are, in reality, likely to (continue to) have far greater immediate impacts on Antarctic ecosystems than climate change per se.

**Fig. 3:**
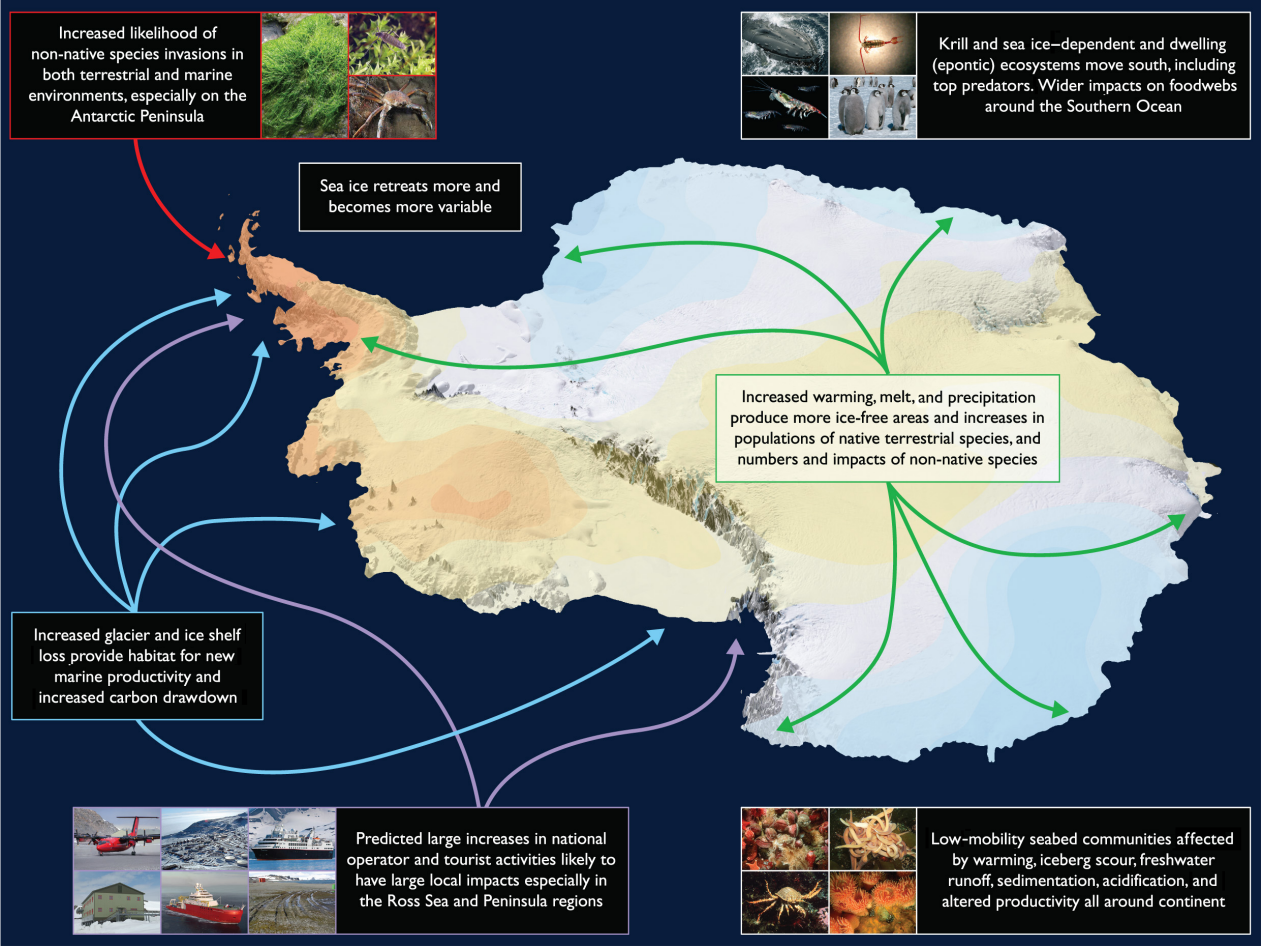
Illustration of the major threats to Antarctic biodiversity in the coming century. Clockwise from top left: Warming reduces ice cover both in the sea and on land, which, combined with increased human activity, makes the establishment of non-native species much more likely (images are the invasive midge *E. murphyi*, and the noted but not established marine seaweed *U. intestinalis* and crab *H. araneus*); the reduction in sea ice and increased variability affects species dependent on sea ice for habitat, notably krill that are a key ecosystem resource for many penguins, seals, and whales [images are humpback whale, krill, copepod (*Calanus propinquus*), and emperor penguin chicks]; low-mobility (and many with limited dispersal) marine species affected by multiple factors, including warming, acidification, freshening, increased sedimentation, etc. [images are brachiopods (*Liothyrella uva*), nemertean worms (*Parborlasia corrugatus*), anemones (*Isotaelia antarctica*), and giant isopod (*Glyptonotus antarcticus*)]; large increases in human activity in terms of more infrastructure, increased tourism, and national field campaigns all directly affect environments on land and sea (images are Dash 7 aircraft, McMurdo station, tourist vessel, Rothera station building, Sir David Attenborough ship, and vehicle tracks on King George Island); reductions in coastal ice make new habitat for new biological productivity in the water column and on the seabed, acting to provide new food for ecosystems and against warming by sequestering carbon; warming, ice melt, and increased precipitation on the continent not only provide new ice-free areas and stimulate increases in populations of native species but also increase likelihood of establishment of non-natives and reduce the isolation and, hence, persistence of native species. Colors on continent show warming and cooling trends over the past 50 years: Red intensity shows warming up to 2°C, and blue shows cooling of up to −1.5°C [following (*195*)].

In terms of conservation management and planning, the instruments and mechanisms are in place to achieve this, both within Antarctica and in the wider global arena, and what is required is the political will and commitment within the Antarctic Treaty System’s signatory nations (*56*, *63*, *195*, *251*). However, it is not yet possible to assess how effective current or future conservation measures are in Antarctica (*43*), especially in the marine environment. The possible use of ex situ conservation has been raised (*56*), and the first Antarctic genetic repository has been established in New Zealand. An important aim must now be to achieve a comprehensive genetic archive of all Antarctic species, so at least their genetic material may be used for societal benefit in future years. Table 1 and Fig. 3 provide overviews of the key features, vulnerabilities, and recommendations for Antarctic marine and terrestrial environments pertinent to consideration of the impacts of environmental change.

**Table 1:** Summary of key features, vulnerabilities, and recommendations for Antarctic marine and terrestrial environments pertinent to consideration of the impacts of environmental change.

**Marine**	**Terrestrial**	**Recommendations**
Most isolated marine environment on Earth, no shelf links to other continents, no water masses flowing to/from other continents through the barrier of the circumpolar current. There is no year-round ice-free intertidal or shallow subtidal habitat. Much higher native biodiversity than expected by area, several groups more diverse than the average for the planet. Crushing predators (e.g., brachyuran crabs, lobsters, and most sharks) very rare to absent. High overall endemism. Gigantism well developed, linked to low metabolic rates and high levels of dissolved oxygen dissolved at low temperatures. Some of the most stable temperatures globally, but other factors among the most variable, e.g., light regime, phytoplankton productivity, and ice cover. Sea temperatures west of the Antarctic Peninsula among the fastest warming globally in the 20th century. Warming predicted to become more widespread around the continent. Biological responses to change vary with the rate of the change, from instantaneous biochemical buffering to migration and evolution; most important immediate responses are acclimatization of physiology through plasticity or genetic adaptation. Biological assemblages developing in areas exposed by glacier and ice shelf retreat (blue carbon) may be the second largest biological response on Earth mitigating warming by sequestering carbon. Many species have poor abilities to cope with warming compared with lower latitude species. Ocean acidification has variable impacts, with some groups such as pteropods negatively affected while others appear resilient to predicted end century acidification. Increased freshwater runoff and lowered salinities, as well as increased sediment input, are expected to have large local impacts especially in fiordic and other coastal systems. High oxygen in cold waters has led to evolution of strong antioxidant defenses. The challenge could lessen with warming. However, warming will increase metabolic costs and reduce available oxygen, likely reducing capacity to raise metabolic rates to do work. In situ experimental manipulations exposing biological communities to predicted end century temperatures for up to 2 years produced unexpected results with greater than expected increases in growth with 1°C of warming and several species showing signs of inability to cope at 2°C of warming. The Southern Ocean was the first to use an ecosystem-based approach to fisheries management, with more sustainable long-term management of living resources than elsewhere. Antarctica contains a repository of global pollution records, increasingly including plastics. Pollution is a concern in marine systems, especially in relation to station sewage outputs. Natural levels of some trace metals from rock erosion may be very high in both marine and terrestrial systems. Viruses from lower latitude sources can infect birds and mammals, and humans are the likely vector. Non-native species invasions in the Antarctic marine environment are currently rare to absent, but warming and loss of ice may allow establishment before the end of the century. Ship traffic has increased 10-fold since the 1960s, with strong regional hot spots where establishment is more likely. Further strong increase expected, with multiple new operator and cruise ships being built.	Continent strongly isolated from lower latitude land by geographical distance, oceanic, and atmospheric circulation. Ice-free ground constitutes tiny proportion of continental area (<0.5%), mostly as “islands” in varying degrees of isolation. Low overall diversity, restricted to microarthropods, microinvertebrates, mostly lower plants, lichens, and microbes. Generally highly endemic biota, with multimillion year or longer presence. Very strong regionalization (ACBRs). Many unknowns remain in terms of lack of survey of many areas or of specific taxonomic groups, meaning “discovery science” is still required. Lack of repeat survey or monitoring restricts ability to detect biodiversity changes. Multiple and highly variable environmental stresses, particularly temperature, desiccation, light/radiation climate, and low nutrients. Liquid water availability is primary driver of biodiversity on the continent. Marine vertebrate nutrient also inputs an important diversity driver in coastal regions and subjects to predicted climate-related changes in vertebrate distribution. Stress tolerance adaptations well developed, in typically “stress-selected” life histories but take up many resources and quid pro quo is that competitive abilities are low. Abiotic variables typically structure biodiversity. Antarctic Peninsula air temperatures among the fastest warming globally in the 20th century, predicted to resume; continent also predicted to face similar warming in next century. Increased precipitation and melt also around the fringes of the continent and Peninsula. At “business-as-usual” warming rates, ice-free area predicted to increase by 25% in next century across entire continent and 300% in Peninsula. Increased area for native and non-native species colonization, and distribution spread, but threat of genetic homogenization. Already well-developed physiological tolerances mean native biota generally not likely to be stressed beyond limits by predicted century-scale changes, although this may occur in specific instances especially considering interactions between multiple variables. Experimental field manipulations generally support these predictions, although representativeness of methodologies has been subject to scrutiny. Continental and peninsula ecosystems to date have suffered relatively little direct human impact, unlike those of sub-Antarctic islands. No extractive or exploitative industries on land. Human presence today limited to national scientific operators and tourism industry. However, multiple direct pressures now increasing, in particular competition for land/land use change, pollution, and inadvertent introduction of non-native species. Rates of anthropogenic introduction already two orders of magnitude or more greater than natural colonization rates. Major, possibly now irreversible, effects of non-native species on several sub-Antarctic islands. Although historical vertebrate introductions have had marked visible effects, contemporary concern relates to invertebrates and plants. Possible step changes or tipping points in ecosystem function in terms of, e.g., predation, pollination, and nutrient turnover. Virtually no knowledge of microbial introductions. Negative impacts of non-native species on Antarctic ecosystems are likely to be greater on a “next-century” time scale than those of other aspects of environmental change. Major station and infrastructure (re)construction programs from multiple national operators, particularly in Victoria Land and Antarctic Peninsula regions.	Achieve a comprehensive genetic archive of all Antarctic species, including microbial, so at least their genetic material may be used for societal benefit in future years. Given the poor resistance capacities of Southern Ocean biota in particular, ex situ conservation measures should be encouraged through gene banks that screen and store the DNA sequences of as many species as can be obtained. Environmental change, genetic homogenization, and direct human impacts (particularly non-native species introductions) present urgent conservation challenges to the Antarctic Treaty Parties requiring timely action and delivery of an effective conservation strategy for both land and ocean. Baseline survey and research are still required to properly document and describe Antarctic biodiversity, with the widespread establishment of ongoing monitoring of natural ecosystems backed by appropriate taxonomic expertise to detect and then investigate changes. There is also an urgent need for higher levels of monitoring and research to identify species and ecosystems that are vulnerable to change, to both predict future outcomes and also to ensure that the best conservation practices are used. There remains a need to link large-scale studies of changes in physical climate with monitoring and identification of change trends (if any) at biologically relevant scales, for instance, as proposed by the SCAR ANTOS (Antarctic Terrestrial and Nearshore Observing System; www.scar.org/science/antos/home/). There is a prescient need for commitment to long-term and multidisciplinary evaluations of environmental change and the responses of the biota in terms of their distributions, physiologies, population genetic modification, and community and ecosystem structure and function. Increased emphasis is required in experimental studies to the inclusion of multiple interacting stressors, realistic timescales of exposure and rates of change, and multiple ecosystem elements, in studies attempting to clarify or predict biological responses. Avoiding and mitigating the impacts of direct human activities requires organizational and personal commitment to active education and adherence to existing procedures and advice; this inherently requires appropriate investment in monitoring (increasingly through remote sensing) of both impacts and recovery. Greater recognition is required of the combination of climate similarity and human operational connectivity between the different biogeographic regions within Antarctica, which further compounds the risk of human-assisted introduction of regionally non-native species. Of the currently known non-native species established in Antarctica (including the sub-Antarctic) since the mid-20th century, virtually all can most plausibly be linked with national operations. Education and awareness are therefore required of the major sources of risk and their mitigation measures, with commitment to investment in monitoring and effective rapid response protocols in place in the event of future transfer events. Greater awareness of and adherence to appropriate and stringent biosecurity procedures are required at both operator and personal individual levels; compared with other continents, the numbers of gateway departure and arrival ports, vessels and aeroplanes, quantities of cargo, and individuals involved make this tractable in terms of applying these measures effectively. Develop means of assessing how successful conservation measures are currently in the Antarctic marine environment, including programs to collect the required data.
